# Synthesis and Evaluation of Bakuchiol Derivatives as Potential Anticancer Agents

**DOI:** 10.3390/molecules23030515

**Published:** 2018-02-23

**Authors:** Cheng-Zhu Wu, Da-Chuan Liu, Xing Guo, Yiqun Dai, Tao Ma, Hong-Mei Li, Qiang Huo

**Affiliations:** School of Pharmacy, Bengbu Medical College, 2600 Donghai Road, Bengbu 233030, China; wuchengzhu0611@foxmail.com(C.-Z.W.); dcliu1226@outlook.com (D.-C.L.); guoxing7010@163.com (X.G.); daiyiqun25@126.com (Y.D.); matao1992@foxmail.com (T.M.)

**Keywords:** bakuchiol derivatives, semi-synthesis, cytotoxicity, migration, invasion

## Abstract

A series of bakuchiol derivatives were synthesized and evaluated for their anti-proliferative and the inhibitory activities on SMMC7721 cell line migration using PX-478 as a positive control. The results showed (*S*,*E*)-4-(7-methoxy-3,7-dimethyl-3-vinyloct-1-en-1-yl)phenol (**10**) to have the best activity among the tested compounds, which included PX-478. In addition, compound **10** showed greater inhibitory activity than that of bakuchiol in the transwell migration and invasion assays at every dose. In western blotting tests, compound **10** showed a promising ability to downregulate the expression of HIF-1α and its associated downstream proteins MMP-2 and MMP-9. Moreover, this effect was dose-dependent and could represent a possible mechanism of action for the anticancer activity of compound **10**.

## 1. Introduction

As sources of new drugs natural products of plant origin play one of the most important roles in the drug research and development field. Bakuchiol (**1**) is an isoprene terpenoid isolated from the seeds of *Psoralea corylifolia* L. (Fabaceae). Since its discovery, bakuchiol has exhibited various biological properties, including anticancer [1,2,3,4], antioxidant [5], estrogenic-like [6,7], antimicrobial [8], antifungal [9], liver protective [10], human carboxylesterase 2 (hCE2) inhibitory [11], and immune-suppressive activities [12]. This evidence suggests that bakuchiol has potential for further development and application.

A few studies have reported the semi-synthesis of bakuchiol and its various activities [1,12,13,14]. In our previous studies, we demonstrated the inhibitory effect of bakuchiol on hypoxia-inducible factor 1α (HIF-1α), with an IC_50_ value of 6.1 μM, in a screening experiment [15]. HIF-1α is an important transcription factor that induces hypoxic conditions. It plays a dominant role in the expressions of correlative genes and is involved in the proliferation, migration, invasion, vascularization, and glycometabolism of tumor cells. Therefore, HIF-1α inhibitors are considered potential therapeutic agents for cancer [16,17].

In this study, the benzene ring, hydroxyl group, and side-chain of bakuchiol (**1**) were modified to obtain a series of bakuchiol derivatives, and their pharmacological activities inhibiting migration and invasion of SMMC7721 cells was evaluated in transwell assays under CoCl_2_-induced hypoxic conditions. Their ability to inhibit the expression of relevant proteins, including HIF-1α and its associated downstream proteins, was evaluated by western blotting.

## 2. Results and Discussion

### 2.1. Chemistry

**Scheme 1:**
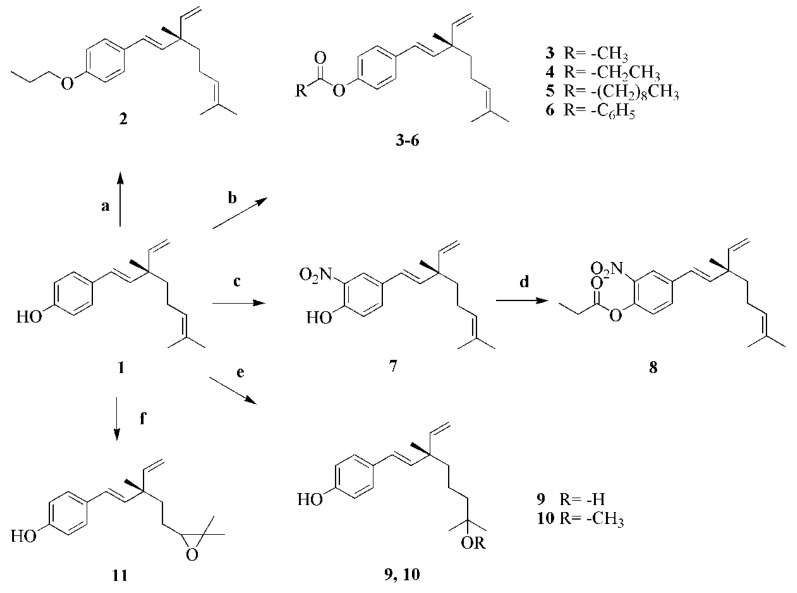
Synthetic routes for bakuchiol derivatives. *Reagents and conditions*: (**a**) bromopropane, K_2_CO_3_, acetone, reflux, 8 h. (**b**) acetic anhydride, propionic anhydride, decyl chloride or benzoyl chloride, catalytic amount of DMAP, methylene chloride, rt. (**c**) nickel nitrate, catalytic amount of *p*-TSA, acetone, reflux. (**d**) propionic anhydride, catalytic amount of DMAP, acetone, rt. (**e**) dilute sulphuric acid, H_2_O or methanol, rt. (**f**) *m*-CPBA, methylene chloride, 0 °C to rt, 0.5 h. rt, 3 h. NaHSO_3_, 0.5 h.

Bakuchiol (**1**) was extracted and isolated from the seeds of *P. corylifolia* L. and purified by silica gel column chromatography. The other target compounds **2**–**11** mentioned in this research were synthesized from **1** in one- or two-step reactions according to the general pathway outlined in Scheme 1. Compound **2** was synthesized from compound **1** by an alkylation reaction using bromopropane, with anhydrous sodium carbonate in acetone as the catalyst. Compounds **3**–**6** were obtained by esterification of compound **1** using acetic anhydride (compound **3**), propionic anhydride (compound **4**), decyl chloride (compound **5**), and benzoyl chloride (compound **6**), respectively, with DMAP as the catalyst. Because the acyl chlorides are unstable, decyl chloride and benzoyl chloride should be used immediately after the compounds are obtained. Compound **7** was obtained by nitration of compound **1** with nickel nitrate in acetone, using *p*-TSA as catalyst. Further, compound **7** was reacted with propionic anhydride to obtain compound **8**. Bakuchiol was reacted with H_2_O and methanol to obtain compounds **9** and **10**, respectively, and with 3-chloro- peroxybenzoic acid to form compound **11**. The detailed synthetic processes are described in the Materials and Methods section. The compounds **2**–**11** were purified by silica gel column chromatography and their chemical structures were characterized using ^1^H-NMR, ^13^C-NMR, ESI-MS and (HR)-ESI-MS.

### 2.2. Anti-proliferative Activity

All compounds were evaluated for their cytotoxicity in vitro against four human cancer cell lines using MTT assays, with ADM (adriamycin) and PX-478 (*S*-2-amino-3-[4′-*N*,*N*,-bis-(2-chloroethyl)amino]phenylpropionic acid *N*-oxide dihydrochloride) as positive control. PX-478 is an inhibitor of constitutive hypoxia-induced HIF-1α and thus HIF-1α transcription factor activity in human tumor xenografts and has marked antitumor activity against larger tumor xenografts, which has a positive correlation with HIF-1α levels [18]. The MTT assays indicated cancer cell viability in response to the different compounds, and the data reflected the inhibition effect of the bakuchiol derivatives on cancer cell proliferation. Based on the results of the MTT assays in Table 1, most of compounds showed cytotoxic activity against various cancer cell lines. Compound **1** exhibited significant cytotoxic activity against four cancer cell lines, however, the inhibition activity almost disappeared when the 4-hydroxy group was alkylated by propane (compound **2**). Among the 4-acylates, compound **3** showed the best cytotoxic activity, while compounds **4** and **5** exhibited reduced activities as the carbon chain length gradually increased, and compound **6**, replaced by benzoyl chloride, only showed little inhibitory effect on SW480. The nitration product of bakuchiol (compound **7**) also exhibited good cytotoxic activity, which was better than its propionyl derivative (compound **8**), which had an IC_50_ of more than 100 μM for inhibition of HepG2. Compounds **9** and **11** also showed commendable activities in MTT assays. Among all the compounds assayed, compound **10** exhibited the best inhibition activity with a low IC_50_. It is worth mentioning that eight compounds, compounds **1**, **3**, **4**, **7**, **8**, **9**, **10**, and **11**, exhibited better activities when compared with the PX-478, on human liver carcinoma SMMC7721 cells.

### 2.3. Preliminary Screening of the Inhibitory Effect on SMMC7721 Migration

**Figure 1:**
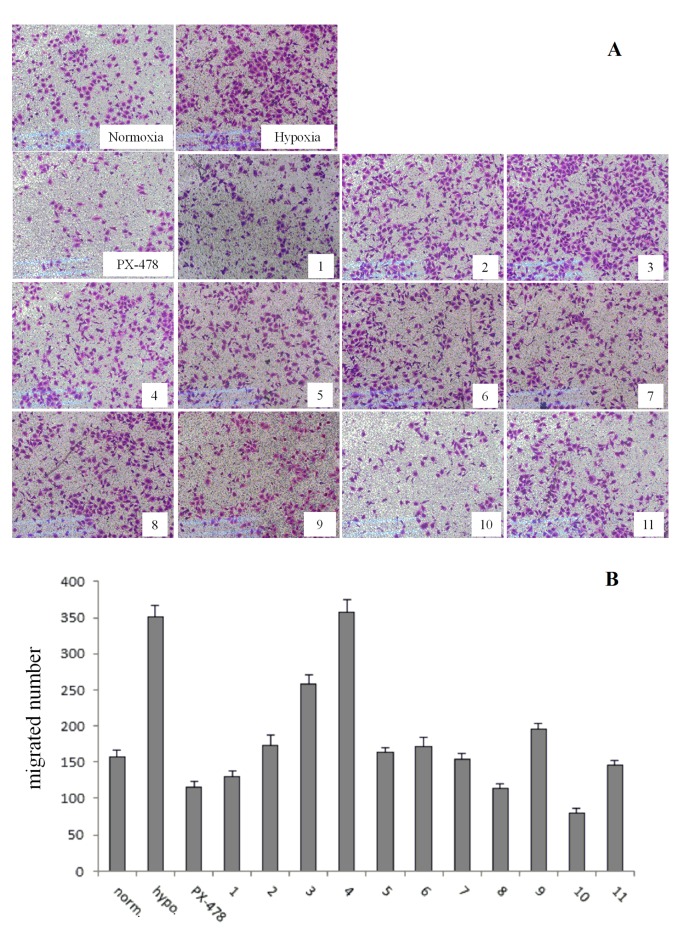
Preliminary screening of the effects on migration of SMMC7721 cells under hypoxia conditions of compounds **1**–**11** (20 μM) compared with positive control (PX-478, 25 μM) by transwell assays. Hypoxia culture condition was established by 150 μM CoCl_2_ induction for 24 h. 1 × 10^5^ cells were seeded into the transwell migration chamber and exposed to compounds for 24 h. (A) The migrated cells at the lower side of the membrane were then stained using Crystal Violet. (B) The quantification analysis. Column, mean; bar, standard deviation; *n* = 3.

Compound **1** and its derivatives **2**–**11** were screened, with PX-478 as the positive control, using transwell assays, which enables us to identify the compounds that inhibit cancer cell migration in vitro. The following observations were obtained and are listed in Figure 1. The preliminary pharmacological screening revealed that three compounds (**1**, **8** and **10**) exhibited the best inhibitory activities on the migration of SMMC7721. Compounds **1** and **8** were almost equivalent to the PX-478; it is also worth mentioning that compound **10** had the best anti-migration activity in the series of derivatives, and was found to be better than PX-478. Because compound **8** could not inhibit all kinds of cancer cells in the MTT assay, before, we selected only compounds **1** and **10** for further experiments.

### 2.4. Ability to Inhibit Cell Migration and Invasion at Various Concentrations of Compounds ***1*** and ***10***

**Figure 2:**
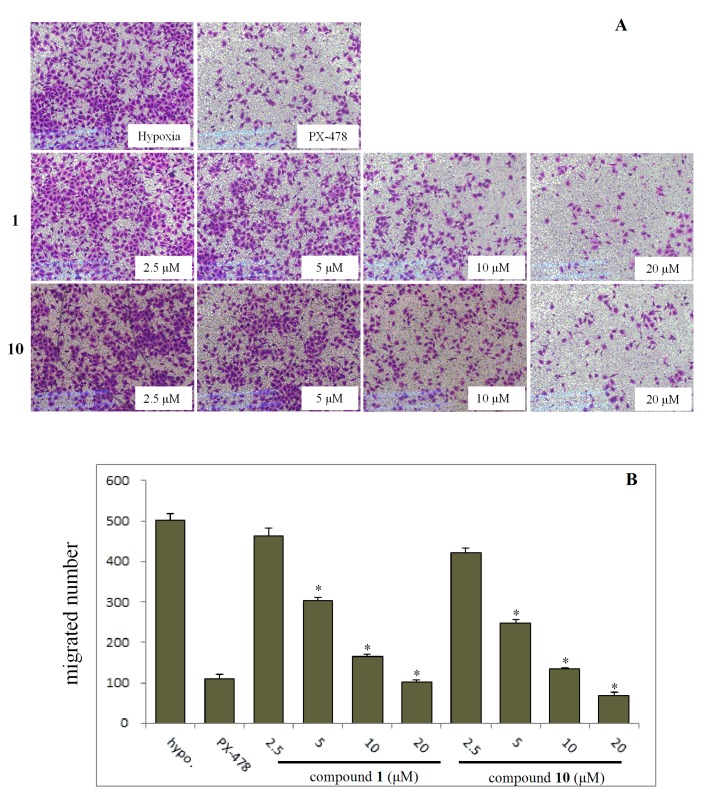
Effects of selected compounds **1** and **10** under the lower concentration gradient with positive control (PX-478) on migration of SMMC7721 cells under hypoxia conditions by transwell assays. 1 × 10^5^ cells were seeded onto the transwell migration chamber and exposed to compounds for 24 h. (**A**) The migrated cells at the lower side of the membrane were then stained using Crystal Violet. (**B**) The quantification analysis. Column: mean; bar: standard deviation; *n* = 3. *p < 0.05 compared with the control.

**Figure 3:**
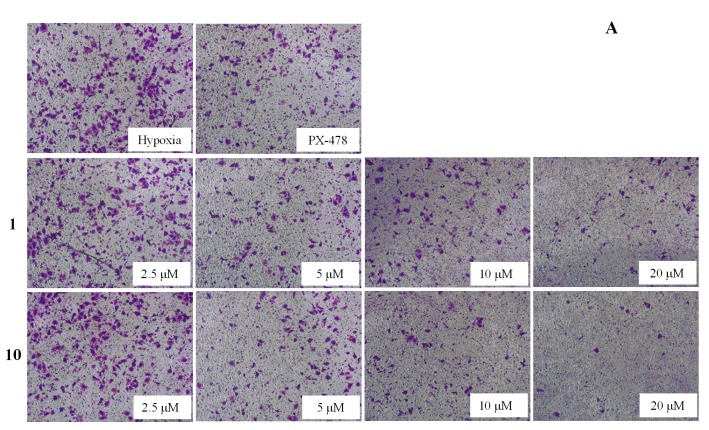

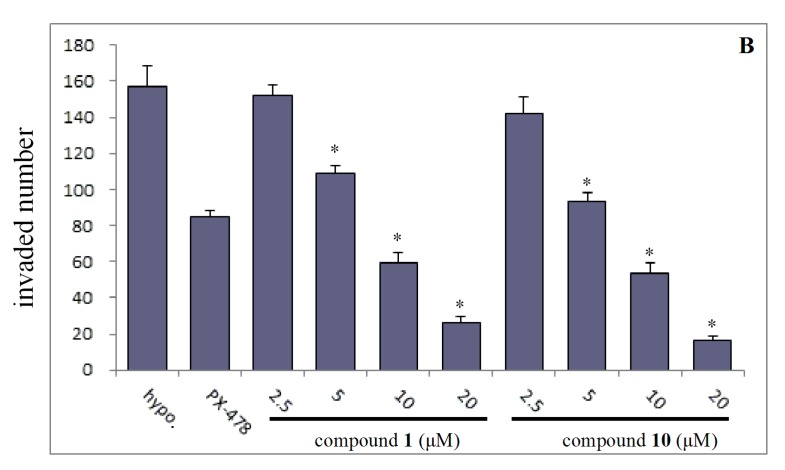
Effects of compounds **1** and **10** under the lower concentration gradient with positive control (PX-478) on invasion of SMMC7721 cell under hypoxia conditions by transwell assays. 1.5 × 10^5^ cells were seeded into the matrigel coated (matrigel:DMEM = 1:8) transwell invasion chamber and exposed to compounds for 36 h. (**A**) The invaded cells at the lower side of the membrane were then stained using Crystal Violet. (**B**) The quantification analysis. Column: mean; bar: standard deviation; *n* = 3. *p < 0.05 compared with the control.

On the basis of the preliminary screening results, the cells were incubated with compounds **1** and **10** in various concentrations to investigate the effect of both inhibiting cell migration (Figure 2) and invasion (Figure 3). The results showed that the migration and invasion abilities of the SMMC7721 cell line were suppressed by compounds **1** and **10** in a dose-dependent manner. In other words, as the concentration was decreased, the anti-migratory and anti-invasive effects of the compounds was reduced too. Both compounds **1** and **10** showed strong anti-migratory activity and anti- invasive activity at 10 and 20 μM, which were better than that exhibited by the positive control, PX-478 (at a dose of 25 μM). The compounds also showed commendable activities when the dose was reduced to 5 μM. Compound **10**, in particular, showed better anti-migratory and anti-invasive activities when compared with that of compound **1** at every dose of the transwell assays, and continued to show some activity even at a dose to 2.5 μM.

### 2.5. Compound ***10*** Inhibits the Expression of Relevant Proteins

**Figure 4:**
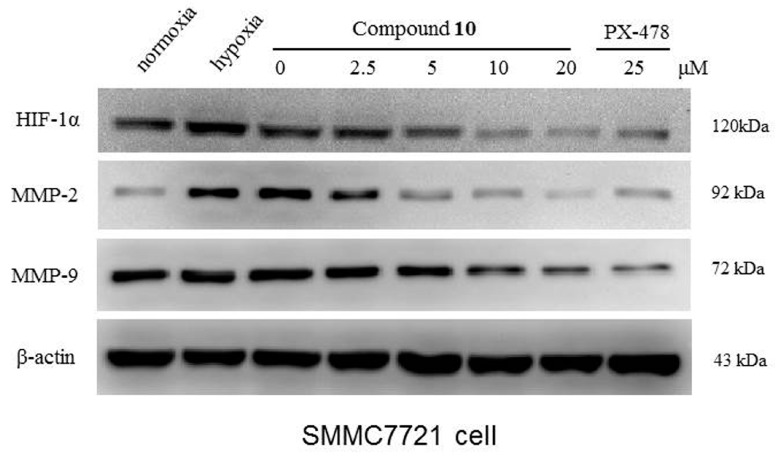
Effects of compound **10** on the expression level of HIF-1α and its associated down- stream proteins: MMP-2 and MMP-9.

Since HIF-1α was reported, its status has grown dramatically, especially in cancer biology [19,20]. Hypoxic conditions are considered as one of the common characteristics of solid tumors [21], which can facilitate tumor metastasis, migration, invasion, and many other important and fatal physiological activities. Therefore, pharmacological inhibition of HIF-1α could be a valuable strategy to modulate tumor apoptotic processes. In this manuscript, we discuss the anticancer activity of compound **10** by testing its ability to inhibit HIF-1α expression with western blotting (Figure 4). 

The western blot results clearly showed that expression of HIF-1α protein was reduced by compound **10** in a concentration-dependent manner under CoCl_2_-induced hypoxic conditions with PX-478 as the positive control. Compared to PX-478, compound **10** showed significant inhibition, with compound **10** showing slightly better inhibition than that shown by compound **1** (Figure S22). Therefore, we continued to study the effect of compound **10** on the expression of MMP-2 and MMP-9 proteins, which are the associated downstream proteins of HIF-1α. We found compound **10** could also inhibit the expression of the two downstream proteins significantly, with PX-478 as the positive control, and its suppression effect was concentration-dependent as well.

## 3. Materials and Methods 

### 3.1. General Information

All reagents used for chemical synthesis were purchased from Sigma-Aldrich (St. Louis, MO, USA). Solvents were distilled before use. All the reactions were monitored by TLC on silica gel F_254_ plates (Qingdao Haiyang Inc., Qingdao, China) for detection of the spot. Silica-gel column chromatography was performed for purification of the products. All NMR spectra were recorded on a DPX 600 instrument (Bruker, Fallanden, Switzerland) using CDCl_3_ as the solvent with TMS as the internal standard. Chemical shifts are expressed in delta (δ) units and coupling constants in Hz. The chemical shifts are reported in parts per million (ppm) and the following abbreviations are used: singlet (s), doublet (d), triplet (t), quartet (q), multiplet (m), and doublet of doublets (dd). Mass spectra were recorded on an ESI-Esquire 3000 instrument (Bruker Daltonics Inc., Billerica, MA, USA). The purity of all compounds (bakuchiol and its derivatives) was determined by HPLC (Shimadzu Co., Tokyo, Japan) and found to be in the 95–99% range.

### 3.2. Isolation of Bakuchiol *(**1**)*

The crude extract of bakuchiol was isolated from the seeds of *P. corylifolia* L. Then the bakuchiol (*S*,*E*)-4-(3,7-dimethyl-3-vinylocta-1,6-dien-1- yl)phenol, **1**, purity > 95%), was purified by silica gel column chromatography and characterized by spectroscopic and chemical methods [15]. R_f_ = 0.45 (*n*-hexane: ethyl acetate = 10:1).

### 3.3. Synthesis of (S,E)-1-(3,7-dimethyl-3-vinylocta-1,6-dien-1-yl)-4-propoxybenzene *(**2**)*

A mixture of bakuchiol (**1**, 256 mg, 1 mmol) and potassium carbonate (276 mg, 2 mmol) in acetone (5 mL) was stirred at reflux temperature for 1 h. Then bromopropane (185 mg, 1.5 mmol) was added, and the mixture was refluxed for another 5 h approximately. After cooling to the room temperature and removing the solution, the reaction was poured in some water and stirred for 0.5 h to wash away the remaining potassium carbonate. Then the solid was obtained by filtered and purified by silica gel column chromatography with 50:1 *n*-hexane:ethyl acetate mixture to obtain compound **2**. The yield was 52.0%. R_f_ = 0.85 (n-hexane: ethyl acetate = 10:1). ^1^H-NMR (CDCl_3_) δ 7.27 (d, *J* = 8.0 Hz, 2H, Ar-H), 6.82 (d, *J* = 8.3 Hz, 2H, Ar-H), 6.25 (d, *J* = 15.9 Hz, 1H, 1-ene-H), 6.04 (d, *J* = 16.3 Hz, 1H, 2-ene-H), 5.87 (dd, *J* = 17.4, 10.7 Hz, 1H, 3-ethenyl-H), 5.10 (t, *J* = 6.4 Hz, 1H, 6-ene-H), 5.05–4.95 (m, 2H, 3-ethenyl-2H), 3.90 (t, *J* = 6.4 Hz, 2H, -OCH_2_-), 2.00–1.88 (m, 2H, -CH_2_-), 1.79 (dd, *J* = 13.9, 6.9 Hz, 2H, -CH_2_-), 1.66 (s, 3H, -CH_3_), 1.57 (s, 3H, -CH_3_), 1.52–1.43 (m, 2H, -CH_2_-), 1.18 (s, 3H, 3-CH_3_), 1.02 (t, *J* = 7.2 Hz, 3H, -CH_3_). ^13^C-NMR (CDCl_3_) δ 158.3, 146.1, 135.7, 131.3, 130.5, 127.1 (2C), 126.6, 124.9, 114.6 (2C), 111.8, 69.6, 42.5, 41.3, 25.7, 23.4, 23.3, 22.6, 17.6, 10.5. ESI-MS: *m/z*, [M + H]^+^ 299.2.

### 3.4. General Procedure for the Synthesis of (S,E)-4-(3,7-dimethyl-3-vinylocta-1,6-dien-1-yl) Phenyl Substituted Carboxylic Esters ***3**–**6***

Compounds **3**–**6** were synthesized from compound **1** with an appropriate alkanoic anhydride or acyl chloride in the presence of a catalytic amount of *N*,*N*-dimethylaminopyridine with 5 mL dichloromethane (DCM) as solvent. Reactions were monitored by TLC. After the reaction was complete, the mixture was poured in ice water and the resulting oily material, which was water insoluble, was separated and dissolved in DCM. Then the organic layer was washed with water for twice, then dried by anhydrous calcium chloride and concentrated under reduced pressure. The acyl derivatives were purified by silica gel column chromatography with a 40:1 *n*-hexane:ethyl acetate mixture as eluent to give compounds **3**–**6**. The yields, R_f_ (*n*-hexane:ethyl acetate = 10:1) and the spectral data of compounds **3**–**6** are given below.

*(S,E)-4-(3,7-dimethyl-3-vinylocta-1,6-dien-1-yl)phenyl acetate* (**3**). The yield was 90.6%. R_f_ = 0.70. ^1^H-NMR (CDCl_3_) δ 7.38–7.32 (m, 2H, Ar-H), 7.04–6.99 (m, 2H, Ar-H), 6.30 (d, *J* = 16.2 Hz, 1H, 1-ene-H), 6.16 (d, *J* = 16.2 Hz, 1H, 2-ene-H), 5.87 (dd, *J* = 17.5, 10.7 Hz, 1H, 3-ethenyl-H), 5.10 (dd, *J* = 7.8, 6.4 Hz, 1H, 6-ene-H), 5.03 (ddd, *J* = 18.7, 14.1, 1.2 Hz, 2H, 3-ethenyl-2H), 2.29 (s, 3H, -COCH_3_), 1.95 (dd, *J* = 16.2, 7.7 Hz, 2H, -CH_2_-), 1.67 (s, 3H, -CH_3_), 1.58 (s, 3H, -CH_3_), 1.53–1.46 (m, 2H, -CH_2_-), 1.20 (s, 3H, 3-CH_3_). ^13^C-NMR (CDCl_3_) δ 169.5, 149.6, 145.7, 138.2, 135.8, 131.4, 127.0 (2C), 126.3, 124.7, 121.6 (2C), 112.1, 42.7, 41.2, 25.7, 23.3, 23.2, 21.1, 17.7. ESI-MS: *m/z*, [M + H]^+^ 299.2.

*(S,E)-4-(3,7-dimethyl-3-vinylocta-1,6-dien-1-yl)phenyl propionate* (**4**). The yield was 61.5%. R_f_ = 0.77. ^1^H-NMR (CDCl_3_) δ 7.35 (d, *J* = 8.6 Hz, 2H, Ar-H), 7.05–6.97 (m, 2H, Ar-H), 6.30 (d, *J* = 16.3 Hz, 1H, 1-ene-H), 6.15 (d, *J* = 16.3 Hz, 1H, 2-ene-H), 5.88 (dd, *J* = 17.5, 10.7 Hz, 1H, 3-ethenyl-H), 5.10 (t, *J* = 7.1 Hz, 1H, 6-ene-H), 5.03 (ddd, *J* = 18.7, 14.1, 1.2 Hz, 2H, 3-ethenyl-2H), 2.58 (q, *J* = 7.5 Hz, 2H, -CH_2_-), 1.95 (d, *J* = 8.3 Hz, 2H, -CH_2_-), 1.67 (s, 3H, -CH_3_), 1.58 (s, 3H, -CH_3_), 1.52–1.47 (m, 2H, -CH_2_-), 1.26 (t, *J* = 7.6 Hz, 3H, -CH_3_), 1.20 (s, 3H, 3-CH_3_). ESI-MS: *m/z*, [M + H]^+^ 313.2.

*(S,E)-4-(3,7-dimethyl-3-vinylocta-1,6-dien-1-yl)phenyl decanoate* (**5**). The yield was 11.7%. R_f_ = 0.80. ^1^H-NMR (CDCl_3_) δ 7.41–7.37 (m, 2H, Ar-H), 7.06–7.02 (m, 2H, Ar-H), 6.34 (d, *J* = 16.2 Hz, 1H, 1-ene-H), 6.19 (d, *J* = 16.2 Hz, 1H, 2-ene-H), 5.92 (dd, *J* = 17.5, 10.7 Hz, 1H, 3-ethenyl-H), 5.15 (td, *J* = 7.1, 3.6 Hz, 1H, 6-ene-H), 5.07 (ddd, *J* = 18.8, 14.1, 1.3 Hz, 2H, 3-ethenyl-2H), 2.58–2.55 (m, 2H, -CH_2_-), 1.99 (dd, *J* = 16.2, 7.6 Hz, 2H, -CH_2_-), 1.79–1.76 (m, 2H, -CH_2_-), 1.71 (s, 3H, -CH_3_), 1.62 (s, 3H, -CH_3_), 1.46–1.40 (m, 4H, -(CH_2_)_2_-), 1.35–1.31 (m, 10H, -(CH_2_)_5_-), 1.24 (s, 3H, 3-CH_3_), 0.93–0.92 (m, 3H, -CH_3_). ^13^C-NMR (CDCl_3_) δ 172.2, 149.9, 145.7, 138.2, 135.7, 131.2, 127.0 (2C), 126.4, 124.9, 121.7 (2C), 112.2, 42.7, 41.4, 34.4, 32.0, 29.9, 29.7, 29.6, 29.5, 29.3, 25.7, 25.1, 23.3, 22.8, 17.6, 14.2. ESI-MS: *m/z*, [M + H]^+^ 411.3.

*(S,E)-4-(3,7-dimethyl-3-vinylocta-1,6-dien-1-yl)phenyl benzoate* (**6**). The yield was 13.9%. R_f_ = 0.70. ^1^H-NMR (CDCl_3_) δ 8.20 (dd, *J* = 8.3, 1.2 Hz, 2H, ph-H), 7.65–7.61 (m, 1H, ph-H), 7.51 (t, *J* = 7.8 Hz, 2H, ph-H), 7.41 (d, *J* = 8.5 Hz, 2H, Ar-H), 7.15 (d, *J* = 8.6 Hz, 2H, Ar-H), 6.34 (d, *J* = 16.3 Hz, 1H, 1-ene-H), 6.19 (d, *J* = 16.3 Hz, 1H, 2-ene-H), 5.89 (dd, *J* = 17.5, 10.7 Hz, 1H, 3-ethenyl-H), 5.12 (t, *J* = 7.2 Hz, 1H, 6-ene-H), 5.04 (ddd, *J* = 18.8, 14.1, 1.3 Hz, 2H, 3-ethenyl-2H), 1.97 (dd, *J* = 16.2, 7.4 Hz, 2H, -CH_2_-), 1.68 (s, 3H, -CH_3_), 1.59 (s, 3H, -CH_3_), 1.52 (dd, *J* = 9.6, 7.8 Hz, 2H, -CH_2_-), 1.22 (s, 3H, 3-CH_3_). ^13^C-NMR (CDCl_3_) δ 165.2, 149.8, 145.7, 138.3, 135.8, 133.6, 131.4, 130.2 (2C), 129.6, 128.6(2C), 127.1 (2C), 126.4, 124.7, 121.7 (2C), 112.1, 42.7, 41.3, 25.7, 23.3, 23.3, 17.7. Its HR-ESI-MS exhibited a pseudo molecular ion peak at *m/z* 361.2162 [M + H]^+^ consistent with the molecular formula C_25_H_28_O_2_.

### 3.5. Synthesis of (S,E)-4-(3,7-dimethyl-3-vinylocta-1,6-dien-1-yl)-2-nitrophenol *(**7**)*

Compound **1** (0.5 mM) was dissolved in acetone (10 mL), then nickel (II) nitrate (1 mmol) and a catalytic amount of *p*-TSA (0.01 mmol) were added. The mixture was refluxed until compound **1** was fully consumed, and then the acetone was removed under vacuum to get the crude product. After extraction with water and DCM, the organic layer was dried with anhydrous calcium chloride overnight and concentrated under reduced pressure. The pure compound **7** was obtained by silica gel column chromatography with *n*-hexane:ethyl acetate mixture (40:1) as eluent. The yield of compound **7** was 51.8%. R_f_ = 0.85 (*n*-hexane:ethyl acetate = 10:1). ^1^H-NMR (CDCl_3_) δ 10.52 (s, 1H, Ar-OH), 8.04 (s, 1H, Ar-H), 7.61 (dd, *J* = 8.3, 1.7 Hz, 1H, Ar-H), 7.10 (d, *J* = 8.7 Hz, 1H, Ar-H), 6.26 (d, *J* = 15.8 Hz, 1H, 1-ene-H), 6.17 (d, *J* = 16.3 Hz, 1H, 2-ene-H), 5.87 (dd, *J* = 17.4, 10.6 Hz, 1H, 3-ethenyl-H), 5.15–5.09 (m, 1H, 6-ene-H), 5.10–4.98 (m, 2H, 3-ethenyl-2H), 1.95 (dd, *J* = 16.4, 8.7 Hz, 2H, -CH_2_-), 1.68 (s, 3H, -CH_3_), 1.58 (s, 3H, -CH_3_), 1.53 - 1.47 (m, 2H, -CH_2_-), 1.21 (s, 3H, -CH_3_). ESI-MS: *m/z*, [M + H]^+^ 302.3.

### 3.6. Synthesis of (S,E)-4-(3,7-dimethyl-3-vinylocta-1,6-dien-1-yl)-2-nitrophenyl Propionate *(**8**)*

Using the preparation and purification described above for compound **4**, and using **7**, propionic anhydride and catalytic amount of *N,N*-dimethylaminopyridine in 5 mL DCM as solvent, compound **8** was prepared in 67.2% yield. R_f_ = 0.50 (*n*-hexane:ethyl acetate = 20:1). ^1^H-NMR (CDCl_3_) δ 8.04 (d, *J* = 2.1 Hz, 1H, Ar-H), 7.59 (dd, *J* = 8.4, 2.1 Hz, 1H, Ar-H), 7.15 (d, *J* = 8.4 Hz, 1H, Ar-H), 6.30 (q, *J* = 16.2 Hz, 2H, 1-ene-2H), 5.87 (dd, *J* = 17.5, 10.7 Hz, 1H, 3-ethenyl-H), 5.09 (dt, *J* = 6.0, 4.8 Hz, 2H, 3-ethenyl-H and 6-ene-H), 5.03 (dd, *J* = 17.5, 1.1 Hz, 1H, 3-ethenyl-H), 2.67 (q, *J* = 7.5 Hz, 2H, -CH_2_-), 1.98 - 1.92 (m, 2H, -CH_2_-), 1.68 (s, 3H, -CH_3_), 1.58 (s, 3H, -CH_3_), 1.52 (ddd, *J* = 8.3, 5.2, 1.7 Hz, 2H, -CH_2_-), 1.29 (t, *J* = 7.5 Hz, 3H, -CH_3_), 1.22 (s, 3H, -CH_3_). ^13^C-NMR (CDCl_3_) δ 172.2, 144.9, 142.6, 141.8, 141.5, 137.1, 131.8, 131.6, 125.2, 124.5, 122.8, 112.8, 42.9, 41.1, 27.5, 25.7, 23.2, 23.1, 17.8, 8.7. Its HR-ESI-MS exhibited a pseudo molecular ion peak at *m/z* 358.2013 [M + H]^+^ consistent with the molecular formula C_21_H_27_NO_4_.

### 3.7. Synthesis of (S,E)-4-(7-hydroxy-3,7-dimethyl-3-vinyloct-1-en-1-yl)phenol *(**9**)* and (S,E)-4-(7-methoxy-3,7-dimethyl-3-vinyloct-1-en-1-yl)phenol *(**10**)*

Compound **1** (0.5 mM, R_f_ = 0.70, DCM:methanol = 50:1) was dissolved in 5 mL methanol, and dilute sulphuric acid (1 mL, 3 M) was slowly added with ice bath cooling. Then the temperature was raised up to 45 °C and the mixture was stirred for 8 h until the reaction was complete (monitored by TLC). After the methanol was removed under reduced pressure, the solid residue was extracted with 250 mL water and the same volume of DCM. Then the organic layer was washed with water, dried over anhydrous sodium sulfate overnight, and concentrated under reduced pressure. The residue was then purified by column chromatography on silica gel, using DCM and methanol mixture (80:1) as eluent, to give compounds **9** and **10**.

*(S,E)-4-(7-hydroxy-3,7-dimethyl-3-vinyloct-1-en-1-yl)phenol* (**9**). The yield was 16.4%. R_f_ = 0.20 (DCM: methanol = 50:1). ^1^H-NMR (CDCl_3_) δ 7.26–7.24 (m, 1H, Ar-H), 7.24–7.23 (m, 1H, Ar-H), 7.10 (s, 1H), 6.77–6.76 (m, 1H, Ar-H), 6.75–6.74 (m, 1H, Ar-H), 6.24 (d, *J* = 16.2 Hz, 1H, 1-ene-H), 6.05 (d, *J* = 16.2 Hz, 1H, 2-ene-H), 5.87 (dd, *J* = 17.5, 10.7 Hz, 1H, 3-ethenyl-H), 5.02 (ddd, *J* = 18.9, 14.1, 1.3 Hz, 2H, 3-ethenyl-H), 1.48–1.44 (m, 4H, -CH_2_-), 1.37–1.33 (m, 2H, -CH_2_-), 1.26 (s, 1H, Ar-OH), 1.20 (s, 6H, 7-CH_3_ and 8-CH_3_), 1.19 (s, 3H, 3-CH_3_). ^13^C-NMR (CDCl_3_) δ 154.9, 146.0, 135.8, 127.4 (2C), 126.6, 116.0, 115.4 (2C), 111.9, 71.4, 44.6, 42.6, 41.9, 29.3 (2C), 23.5, 19.3. ESI-MS: *m/z*, [M − H]^+^ 273.2.

*(S,E)-4-(7-methoxy-3,7-dimethyl-3-vinyloct-1-en-1-yl)phenol* (**10**). The yield was 20.0%. R_f_ = 0.40 (DCM: methanol = 50:1). ^1^H-NMR (CDCl_3_) δ 7.25–7.24 (m, 1H, Ar-H), 7.24–7.23 (m, 1H, Ar-H), 6.78–6.76 (m, 1H, Ar-H), 6.76–6.75 (m, 1H, Ar-H), 6.24 (d, *J* = 16.2 Hz, 1H, 1-ene-H), 6.05 (d, *J* = 16.2 Hz, 1H, 2-ene-H), 5.87 (dd, *J* = 17.5, 10.7 Hz, 1H, 3-ethenyl-H), 5.04–4.98 (m, 2H, 3-ethenyl-2H), 3.16 (s, 3H, -OCH_3_), 1.47–1.41 (m, 4H, -CH_2_-), 1.34–1.28 (m, 2H, -CH_2_-), 1.18 (s, 3H, 3-CH_3_), 1.13 (s, 6H, -CH_3_). ^13^C-NMR (CDCl_3_) δ 155.0, 146.1, 135.8, 130.6, 127.4 (2C), 126.6, 115.4 (2C), 111.9, 75.0, 49.1, 42.6, 42.0, 40.5, 25.0 (2C), 23.5, 18.8. Its HR-ESI-MS exhibited a pseudo molecular ion peak at *m/z* 289.2139 [M + H]^+^ consistent with the molecular formula C_19_H_28_O_2_.

### 3.8. Synthesis of 4-((3S,E)-3-(2-(3,3-dimethyloxiran-2-yl)ethyl)-3-methylpenta-1,4-dien-1-yl) phenol *(**11**)*

Compound **1** (1.5 mmol) was dissolved in DCM (6 mL). 3-Chloroperoxybenzoic acid (*m*-CPBA, 3 mmol) dissolved in 5 mL DCM was added dropwise to the solution under ice bath cooling, and then the mixture was stirred at room temperature for 4.5 h, until the reaction was complete as monitored by TLC. Saturated sodium bicarbonate was added and stirred for 1 h to neutralize the excess *m*-CPBA. After pouring into 250 mL water, the aqueous layer was extracted thrice with 90 mL dichloromethane (30 mL × 3). The combined dichloromethane layers were dried over anhydrous sodium sulfate overnight. Evaporation of the solvent gave a crude product, which was purified by silica gel column chromatography with *n*-hexane:ethyl acetate mixture (10:1) as eluent to give the title compound in 27.4% yield. R_f_ = 0.30 (*n*-hexane:ethyl acetate mixture = 10:1). ^1^H-NMR (CDCl_3_) δ 7.25–7.24 (m, 1H, Ar-H), 7.24–7.23 (m, 1H, Ar-H), 6.78–6.77 (m, 1H, Ar-H), 6.76–6.75 (m, 1H, Ar-H), 6.28–6.24 (m, 1H, 1-ene-H), 6.07–6.01 (m, 1H, 2-ene-H), 5.89–5.83 (m, 1H, 3-ethenyl-H), 5.07–5.02 (m, 2H, 3-ethenyl-2H), 2.71 (t, *J* = 5.7 Hz, 1H, 3′-CH-), 1.58–1.54 (m, 6H, -CH_3_), 1.31–1.28 (m, 2H, -CH_2_-), 1.26–1.24 (m, 2H, -CH_2_-), 1.20 (s, 3H, -CH_3_). ^13^C-NMR (CDCl_3_) δ 154.8, 145.5, 135.2, 129.8, 127.4 (2C), 126.9, 115.4 (2C), 112.2, 64.7, 58.5, 42.2, 37.5, 24.9, 24.2, 23.4, 18.7. ESI-MS: *m/z*, [M + H]^+^ 273.2.

### 3.9. Cell Culture, Growth Conditions, and Treatments

SMMC7721 (human liver carcinoma cells), HepG2 (human liver hepatocellular carcinoma cells) and SW480 (human colorectal carcinoma cells) were cultured in Dulbecco’s modified Eagle’s medium (DMEM), which was obtained from Gibco (Grand Island, NY, USA). MDA-MB-231 cells (human breast cancer cells) were cultured in minimum essential medium (MEM, Gibco, Grand Island, NY, USA) supplemented with 10% fetal bovine serum (FBS, Millipore, Billerica, MA, USA) and maintained at 37 °C in a humidified incubator with 5% CO_2_. All four types of cancer cells were obtained from the Shanghai Cell Bank of the Chinese Academy of Sciences (CAS). To establish hypoxic conditions, the culture medium was supplemented with 150 μM CoCl_2_ for 24 h. The compounds were dissolved in dimethyl sulfoxide (DMSO, BIOSHARP, Hefei, China). PX-478 (Selleck Chemicals, Houston, TX, USA), a selective HIF-1α inhibitor, was dissolved in ddH_2_O. The control group was treated with DMSO only under identical conditions.

### 3.10. MTT Assay 

Cells were seeded in 96-well microtiter plates at a density of 5 × 10^3^ cells per well and treated with different concentrations of the compounds and adriamycin for 48 h. At the end of the incubation period, 15 μL of MTT (purchased from Sigma, St. Louis, MO, USA), at a dose of 5 mg/mL in phosphate-buffered saline (PBS), was added to each well and incubated at 37 °C for 4 h. After 4 h, the MTT solution was replaced with 150 μL of DMSO to dissolve the formazan crystals. The plates were further incubated for 30 min, and the cell viability was determined by measuring absorbance at a test wavelength of 490 nm with the microplate reader (Synergy HT, BioTek, Winooski, VT, USA).

### 3.11. Cell Migration Assay

The migration assay, which was used to screen the active compounds from the target products, was performed using a 24-well cell culture plate with 8.0 μm pore size membrane inserts without matrigel. First, 5 × 10^4^ SMMC7721 cells were added to the upper wells, and the chambers were incubated for 24 h at 37 °C. The lower chamber was filled with 800 μL of 10% FBS as a chemoattractant. After 24 h under normoxic conditions, the cells that had migrated were stained (Crystal Violet) and photographed under a light microscope at 200× magnification. The number of migratory cells was then counted and analyzed to determine statistically significant differences. Each condition was assayed in triplicate, the experiments were performed independently at least three times, and the results were expressed as the number of cells/field. One-way analysis of variance was used to determine significant differences. The assay described here was also used to determine the influence of the compounds **1** and **10** at different concentrations on the migration of SMMC7721 cells.

### 3.12. Cell Invasion Assay

The invasion assay was performed using a 24-well cell culture plate with 8.0 μm pore size membrane inserts. The membrane undersurface was coated with 50 μL of matrigel with serum-free medium for 30 min at 37 °C. The rest of the operational steps and the conditions of the invasion assay were the same as those described for the cell migration assay.

### 3.13. Western Blotting

The SMMC7721 cells were plated in a 6-well culture plate at a density of 5 × 10^5^ cells per well. After incubation with drugs, the cells were lysed with SDS buffer containing 200 mM Tris (pH 6.8), 40% glycerol, and 10% mercaptoethanol. The protein (30 μg) obtained from cell extracts was separated using 10–15% SDS/polyacrylamide gel electrophoresis, and then transferred to a polyvinylidene difluoride (PVDF) membrane. Membranes were blocked with 10% skimmed milk in Tris-buffered saline containing 0.1% Tween-20 (TBST) for 1 h at room temperature, and then incubated overnight at 4 °C with respective primary antibodies [anti-HIF-1α (Epitomics, Burlingame, CA, USA), anti-MMP-2 (Santa Cruz Biotechnology Inc., Santa Cruz, CA, USA), anti-MMP-9 (Santa Cruz Biotechnology Inc., Santa Cruz, CA, USA), and anti-β-actin (Santa Cruz Biotechnology Inc., Santa Cruz, CA, USA)] diluted to 1:1000–2000 in TBST containing 5% skimmed milk. After washing three times with TBST, the membranes were incubated with horseradish peroxidase- conjugated anti-rabbit and anti-mouse serum, which were used as secondary antibodies (diluted 1:10,000 in TBST containing 5% skimmed milk) for 1 h at room temperature. After incubation, the antigen–antibody complexes were visualized using the ECL kit and β-actin was analyzed as a loading control.

### 3.14. Statistical Analysis

All experiments were repeated three times. Results are expressed as the mean ± standard deviation. Differences were evaluated using one-way analysis of variance with the least significant difference post hoc test for multiple comparisons via SPSS (version 17.0, SPSS Inc., Chicago, IL, USA). *p* < 0.05 was considered to indicate a statistically significant difference.

## 4. Conclusions

In summary, a series of bakuchiol derivatives **2**–**11** were synthesized and evaluated in vitro for their anti-proliferative, anti-migratory, and anti-invasive activities. Our data revealed that compound **10** showed the best activity among all the assayed compounds. We would like to mention that this was the first time the compound **10** was synthesized, evaluated, and explored to study its mechanisms, and a more in-depth and comprehensive study to confirm these results would be worthwhile. Further study and evaluation of bakuchiol derivatives has broad prospects and is important in the search for new anticancer agents.

## Figures and Tables

**Table 1:** Anti-proliferative activity of compounds **1**–**11** (IC_50_, μM, 48 h).

Compound	SMMC7721 ^a^	HepG2 ^b^	MDA-MB-231 ^c^	SW480 ^d^
1	15.44 ± 3.91 ^e^	21.18 ± 1.16	12.97 ± 1.22	9.96 ± 0.15
2	>100 ^f^	>100	>100	>100
3	10.65 ± 0.56	16.17 ± 0.06	9.28 ± 1.02	14.66 ± 0.19
4	19.84 ± 0.66	27.95 ± 1.06	20.75 ± 0.91	27.66 ± 0.16
5	46.80 ± 5.20	>100	>100	>100
6	>100	>100	>100	59.88 ± 1.62
7	9.97 ± 0.11	19.06 ± 0.55	25.20 ± 1.44	30.63 ± 1.43
8	12.95 ± 0.60	>100	15.58 ± 0.72	39.94 ± 1.57
9	9.62 ± 0.23	17.42 ± 1.32	26.66 ± 1.14	15.35 ± 0.34
10	8.53 ± 0.88	4.60 ± 0.13	8.79 ± 0.53	5.01 ± 0.42
11	23.26 ± 0.48	7.22 ± 0.70	14.93 ± 0.64	32.78 ± 0.54
ADM ^g^	0.77 ± 0.08	3.05 ± 0.32	2.23 ± 0.05	0.26 ± 0.06
PX-478	27.57 ± 0.45	28.67 ± 0.68	38.63 ± 0.53	35.42 ± 0.39

^a^: SMMC7721: human liver carcinoma cells; ^b^: HepG2: human liver hepatocellular carcinoma cells; ^c^: MDA-MB-231: human breast cancer cells; ^d^: SW480: human colorectal carcinoma cells; ^e^: The half maximal inhibitory concentration ± standard deviation (SD); ^f^: No inhibitory activity at 100 μM; ^g^: Adriamycin.

## Supplementary Materials

The following are available online, Figures S1–S22.
